# Bud Dormancy in Perennial Fruit Tree Species: A Pivotal Role for Oxidative Cues

**DOI:** 10.3389/fpls.2018.00657

**Published:** 2018-05-16

**Authors:** Rémi Beauvieux, Bénédicte Wenden, Elisabeth Dirlewanger

**Affiliations:** UMR 1332 BFP, INRA, Université de Bordeaux, Villenave-d’Ornon, France

**Keywords:** bud dormancy, perennial fruit species, hormonal pathways, carbohydrate metabolism, oxidative stress

## Abstract

For perennial plants, bud dormancy is a crucial step as its progression over winter determines the quality of bud break, flowering, and fruiting. In the past decades, many studies, based on metabolic, physiological, subcellular, genetic, and genomic analyses, have unraveled mechanisms underlying bud dormancy progression. Overall, all the pathways identified are interconnected in a very complex manner. Here, we review early and recent findings on the dormancy processes in buds of temperate fruit trees species including hormonal signaling, the role of plasma membrane, carbohydrate metabolism, mitochondrial respiration and oxidative stress, with an effort to link them together and emphasize the central role of reactive oxygen species accumulation in the control of dormancy progression.

## Introduction

In the context of perennial plants, bud dormancy is a crucial step in the phenology cycle, as its progression over winter determines the quality of bud break, flowering and fruiting. The term “dormancy” is associated with temporary suspension of visible growth. It comprises true dormancy (“rest” or “endodormancy”) triggered by internal factors, and climatic dormancy (“quiescence” or “ecodormancy”) controlled by external factors (Lang et al., 1987; Considine and Considine, 2016). These phases of dormancy are alleviated by different elements: release of endodormancy requires cold accumulation whereas ecodormancy advances with heat accumulation toward bud break. For perennial fruit species, in the context of global warming, endodormancy release may be a critical step in the future due to insufficient chill accumulation, directly affecting flowering quality and uniformity, and thus leading to a drastic reduction of fruit production. In the past decades, much work has been done to unravel the mechanisms underlying dormancy period, and the diversity of approaches used is indicative of the complexity of the trait. Early studies at the beginning of the 20th century mainly dealt with the observation of the phenomenon itself and the effects of dormancy alleviating molecules. In the 70s, advances in microscopy and subcellular techniques allowed novel observations of cellular modifications over the dormancy period. Later, between 1980 and 2000, physiological studies including metabolic analyses led to further description of the main pathways involved, more recently highlighted by genetic and genomic studies. Numerous studies, notably transcriptomic analyses, have led to the identification of common molecular pathways regulating bud dormancy in trees (Rohde et al., 2007; Ruttink et al., 2007; Yamane et al., 2008; Jiménez et al., 2010; Leida et al., 2010; El Kayal et al., 2011; Liu et al., 2012; Bai et al., 2013; Zhong et al., 2013; Xu et al., 2016; Tarancón et al., 2017). Specific gene expression patterns over the course of dormancy featured hormone signaling, carbon metabolism, stress response and chromatin modification (Regier et al., 2010; Rios et al., 2014; Saito et al., 2015; Wisniewski et al., 2015; Wen et al., 2016). Among the main pathways identified, the *Dormancy-Associated MADS-BOX* (*DAM*) genes have been a constant interest since they were proposed to cause the non-dormant phenotype of the *evg* mutant of peach (Rodriguez et al., 1994; Bielenberg et al., 2004, 2008; Howe et al., 2015). In peach, the six tandem-arrayed *DAM* genes display distinct seasonal patterns with peaks in expression at different times during dormancy (Li et al., 2009), supporting their role in promoting and maintaining dormancy (Jiménez et al., 2010; Hao et al., 2015). Following this finding, *DAM* genes and their involvement in dormancy have been extensively studied in perennial plants including leafy spurge (Horvath et al., 2008; Anderson et al., 2010), apple (Mimida et al., 2015), Japanese pear (Ubi et al., 2010; Saito et al., 2013), tea plant (Hao et al., 2017), Kiwifruit (Wu et al., 2017), and Japanese apricot (Yamane et al., 2008; Sasaki et al., 2011). In poplar, genes homologous to *CONSTANS* (*CO*) and *FLOWERING LOCUS T* (*FT*) have key roles in the control of dormancy (Böhlenius et al., 2006; Hsu et al., 2011; Srinivasan et al., 2012) and the chill-induced release of endodormancy (Rinne et al., 2011). Recent reviews have nicely described the molecular advances in dormancy studies (Cooke et al., 2012; Rios et al., 2014; Sanchez-Perez et al., 2014) therefore we will not focus our attention on these aspects. Overall, all these pathways are interconnected in a very complex manner and so far no integrative scenario has been proposed to precisely describe their interactions. Nonetheless one particular pathway seems central and is almost always highlighted in the recent studies: the response to oxidative stress and the reactive oxygen species (ROS). Notably, most of the studies using dormancy release substances such as HC (Hydrogen Cyanamide) show they induce the ROS scavenging systems. Interestingly, ROS are also thought to be a key signal during plant development for many aspects including dormancy (Considine and Foyer, 2014), as shown by their link to the hormonal interplay, cell wall loosening, and ion channels in seeds. Here, we review both early and recent findings on the dormancy processes in buds of temperate fruit trees species including hormonal signaling, the role of plasma membrane, carbohydrate metabolism, mitochondrial respiration and oxidative stress, with an effort to link them together and emphasize the central role of ROS accumulation in the control of endo- and ecodormancy progression.

## Oxidative Stress and Redox Cues

**FIGURE 1:**
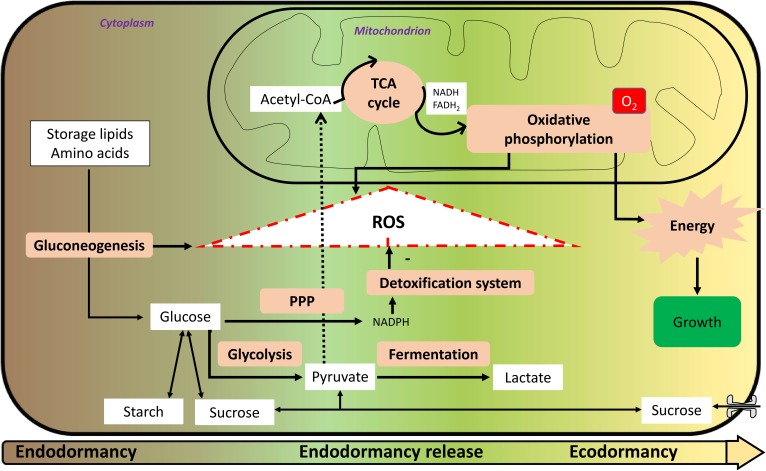
Metabolism and reactive oxygen species (ROS) formation from endodormancy until ecodormancy. From endodormancy to ecodormancy, metabolism is of primary importance. Storage lipids and amino acids are degraded through the gluconeogenesis pathway to yield glucose. Glucose car be interverted to starch and/or sucrose. It can also be degraded through the glycolysis pathway to yield pyruvate that will migrate to the mitochondria and will be metabolized through the tricarboxylic acid (TCA) cycle to enable oxidative phosphorylation to occur with reducing power in the form of nicotinamide adenine dinucleotide (NADH) and flavine adenine dinucleotide (FADH_2_). This progressive reactivation of oxidative phosphorylation during endodormancy release is partly responsible for ROS formation with gluconeogenesis. Glucose may also be oxidized *via* the pentose phosphate pathway (PPP) to yield reducing power in the form of nicotinamide adenine dinucleotide phosphate (NADPH), enabling the detoxification system to operate. During endodormancy release period, there is a net positive accumulation of ROS that trigger mechanisms of endodormancy release. During ecodormancy, oxidative phosphorylation is more efficient and enable energy production and mechanisms of growth that are necessary for bud bursting and flowering.

In contrast to chilling-induced breaking of dormancy, exposing dormant buds to sub-lethal freezing and high temperatures as well as other sub-lethal treatments for a short period of time can overcome rest relatively rapidly (Orffer and Goussard, 1980; Nee and Fuchigami, 1992). Following these observations, numerous lines of evidence have highlighted that stresses, especially oxidative and respiratory stresses, are involved in the release of buds from dormancy. These stresses trigger the production of ROS, which have been shown to be critical throughout plant life and development (Considine and Foyer, 2014). This production of ROS including H_2_O_2_ in buds suggests that they may act as key signaling molecules for dormancy release (Kuroda et al., 2002, 2005; Pérez and Burgos, 2004; Prassinos et al., 2011; Vergara et al., 2012; Hussain et al., 2015; Tan et al., 2015). These hypotheses are further supported by observations that exogenous H_2_O_2_ can substitute for chilling, thus confirming that an increase in H_2_O_2_ levels may activate the sequence of reactions involved in the breaking of bud dormancy (Pérez and Burgos, 2004; Kuroda et al., 2005; Pérez et al., 2008). Conversely, treatment of potato tuber with a NADPH oxidase inhibitor leads to decreased ROS production and delayed dormancy release (Liu et al., 2017). In plant cells, ROS are continuously produced as a consequence of aerobic metabolism in all the intracellular organelles. Cells have the capacity to rapidly produce and scavenge different forms of ROS levels, as a result of a balance between formation and detoxification rates, with a tight link to cellular metabolism, making ROS good signals to monitor changes in cellular metabolism (Mittler et al., 2011). Another indication that oxidative stress is an important part of the process of dormancy is that antioxidant defense and detoxification pathways are upregulated during dormancy release, including catalase (CAT), glutathione peroxidase (GR), superoxide dismutase (SOD), ascorbate peroxidase (APX) and peroxidase superfamily proteins (Scalabrelli et al., 1991; Or et al., 2000; Halaly et al., 2008; Leida et al., 2010; Prassinos et al., 2011; Vergara et al., 2012; Viti et al., 2012; Bai et al., 2013; Zhuang et al., 2013; Guzicka et al., 2017). This is especially true for buds treated with dormancy-breaking compounds. In apricot flower buds, gibberellic acid 4 (GA_4_) treatment upregulates oxidation-reduction proteins and the authors hypothesized that GA_4_ application led to the development of oxidative stress and to subsequent dormancy release (Zhuang et al., 2013). HC has been widely used by growers to overcome low and uneven bud break and the mechanisms that underlies its dormancy-breaking effect is extensively studied in fruit species. Many studies show that a significant increase in H_2_O_2_ levels is the main metabolic change produced by HC, often linked to an inhibition of CAT activity (Bartolini et al., 1996; Pérez and Lira, 2005; Halaly et al., 2008; Pérez et al., 2009; Tan et al., 2015). However, recent genomic studies show that a wide range of genes is differentially regulated after HC application such as genes related to cell wall loosening, hormonal response, carbohydrate and protein metabolism (Ophir et al., 2009; Pérez et al., 2009; Liu et al., 2015; Sudawan et al., 2016; Ionescu et al., 2017) thus linking oxidative stress, mitochondrial activity, hypoxia, cytokinins, auxin, jasmonate and ethylene signaling pathways to HC-induced dormancy release. Based on these findings, HC application is thought to trigger transient oxidative stress and activate detoxification systems. Subsequently, most pathways proposed to be involved in dormancy release are activated: degradation of callose, inhabitation of abscisic acid (ABA), GAs, glycolysis, cytokinins (Ophir et al., 2009; Pérez et al., 2009; Zheng et al., 2015; Ionescu et al., 2017). Another clue for the involvement of ROS during dormancy is the response of several ROS scavenging systems that were closely analyzed during bud dormancy. The glutathione and ascorbate pathways are crucial for the detoxification of H_2_O_2_. The overall content of glutathione was shown to increase in concomitance with the endodormancy overcoming. Moreover, the ratio between reduced (GSH) and oxidized (GSSG) glutathione, was reported to be associated to the dormancy stage: higher levels of GSH at the end of rest compared to beginning of the phase (Siller-Cepeda et al., 1992; Wang and Faust, 1994; Kocsy et al., 2001; Bartolini et al., 2006). This ratio has been shown to be under the control of the glutathione reductase (GR) activity in Japanese pear (Zanol et al., 2010). In dormant grapevine buds, HC upregulates genes involved in ascorbate, glutathione and pentose phosphate pathway (PPP) detoxification pathways (Pérez et al., 2009; Sudawan et al., 2016). Temporary induction of the PPP in response to oxidative stress may provide a way to recharge the system with NADPH for detoxification through the ascorbate/glutathione system (**Figure 1**). Such an induction has been observed in apricot buds after a treatment with GA_4_ (Zhuang et al., 2015), thus allowing the production of NADPH in the absence of mitochondrial respiration. All these studies provide evidence that ROS play a crucial role during dormancy and raise the questions of the mechanism involved in the oxidation system, especially how other pathways interact or are directly controlled by oxidative cues.

## Mitochondrial Respiration

Mitochondrial respiration is the primary cellular source of ROS on non-photosynthetic tissues in the context of healthy conditions (Van Aken et al., 2009), whereas plasma membrane and cell-wall NADPH/NADH oxidases are major producers in the context of defense response (Davies et al., 2006). An elevation of ROS production is noted in abnormal conditions, such as hypoxia or hyperoxia (Turrens, 2003). During dormancy, hypoxia and the inhibition of mitochondrial respiration can be responsible for the increase in ROS content observed in dormant buds (Vergara et al., 2012; Meitha et al., 2015, 2018), potentially by activating gluconeogenesis, and therefore enhances grape bud dormancy release (Ophir et al., 2009; Rubio et al., 2014; Sudawan et al., 2016). Likewise, O_2_ deprivation raises glycolysis and ethanolic fermentation which could lead to the production of ROS (Pérez et al., 2009). Moreover, treatment of isolated grape bud mitochondria with sodium azide, another dormancy release molecule, inhibited O_2_ uptake (Pérez et al., 2009) and mitochondria under hypoxia have been shown to have less TCA cycle enzyme activities and reduced ATP production in maize and potato (Considine et al., 2017). In response to shortening photoperiod and low temperature, respiration may be impaired as part of the growth cessation and dormancy onset processes. For example, ABA, which participates to dormancy maintenance, has been shown to inhibit certain isoforms of the tricarboxylic acid (TCA) cycle isozymes in floral buds of peach (Oncelay et al., 1979) or sucrose transporters in vine (Murcia et al., 2015), thus comforting the hypotheses that respiration processes are affected during dormancy. All these elements suggest that a respiratory stress must be involved in the release of buds from dormancy through abnormal positive net production of ROS.

Regulation of respiration is central to the transition from rest to metabolically active state, generating the ATP needed for cell functioning and growth. In aerobic respiration, mitochondria carry out the final steps of this process and generate the bulk of ATP through (i) the TCA cycle, (ii) the oxidative phosphorylation electron chain, (iii) the alternative oxidase (AOX), and (iv) the set of carriers and channels that provide the substrates and cofactors from the cytosol. Respiration rate and depth of dormancy were shown to be inversely related in grapevine buds (Parada et al., 2016), associated with contrasted response to temperature and glucose, two stimuli that normally increase respiration in plant tissues. While respiration in non-dormant buds rose sharply in response to both stimuli, respiration in dormant buds was only slightly affected, thus suggesting that respiration is inhibited in dormant buds. Several processes can explain this repression of mitochondrial respiration during dormancy. Firstly, some studies report that mitochondria activity might be altered over dormancy progression, with modifications in their number (Felker et al., 1983) or their structure (Guzicka et al., 2017), and could be linked to availability of oxygen and requirements of oxidative phosphorylation (Considine et al., 2017). Thereafter, respiration in dormant bud cells might be affected. Secondly, as described before, dormant cells are subjected to carbon starvation and repression of cell-to-cell transport, coupled with bud scales that have low oxygen permeability. Analyses of gene expression and O_2_ pressure measurements suggest that dormant buds reside in a hypoxic state and return to the oxygenated state during bud burst (Meitha et al., 2015). According to experiments on O_2_ consumption and CO_2_ production of grapevine twigs in hypoxic conditions (solution of chlorpromazine) or normal conditions, fermentation pathway has been suspected to be involved in dormancy release (Pouget, 1965). Recent findings on transcript abundance of key genes such as lactate dehydrogenase or alcohol dehydrogenase tend to confirm the activation of the fermentation pathway in dormant bud cells under chilling or dormancy-breaking reagent treatments (Or et al., 2000; Halaly et al., 2008; Ophir et al., 2009; Pérez et al., 2009), which is characteristic of low-oxygen conditions. Furthermore, plasma membrane properties are modified during dormancy and we hypothesize that chill modifies the properties of membrane-bound proteins, as was shown for the succinate oxidase activity for mitochondria of Jerusalem artichoke tubers (Chapman et al., 1979): the Arrhenius activation energy was high during dormancy and decreased at the termination of dormancy. Finally, it has been proven that the level of ATP or the ATP/ADP ratio change over dormancy progression: low levels of ATP are characteristic of endodormancy while a steep rise in ATP/ADP ratio marks the end of ecodormancy (Bonhomme et al., 2000). The mitochondrial ATP synthase complex requires inorganic phosphate delivery by the mitochondrial phosphate transporter (MTP). The expression level of *MPT* is low during dormancy and up-regulated to promote respiratory rate and energy metabolism for bud dormancy release in tree peony (Huang et al., 2008; Zhang et al., 2016). These results are consistent with the hypothesis that phosphate is compartmentalized during dormancy (Bonhomme et al., 2000) leading to the inhibition of respiration and ATP production.

According to all these knowledge, the role of the mitochondrial respiration during the different phases of dormancy is proposed in **Figure 1**: during endodormancy, hypoxia and the inhibition of mitochondrial respiration can be responsible for the increase in ROS content that reach a maximum at the endodormancy release, and at this stage, recovery of mitochondrial respiration during the ecodormancy period.

## Carbohydrates Metabolism

In addition to the role of mitochondrial respiration toward positive ROS production, carbohydrate metabolism seems to be crucial for ROS systems. During dormancy, a carbon starvation is noted, and glucose is a key molecule. It may be produced *via* storage molecules such as lipids, notably through beta-oxidation and neoglucogenesis, responsible for a net ROS production (Dieuaide et al., 1992). On one hand, glucose may be processed in the glycolysis and then metabolized in the mitochondria for ROS production; on the other hand, it may be processed through the PPP to yield reducing power and participate to ROS detoxification. For example, in yeast, it has been shown that low glucose amounts induce a decreased mitochondrial ROS production (Barros et al., 2004). Interestingly, it has been shown that a switch from glycolysis to PPP during germination in *Arabidopsis* seed is a scavenging system for oxidative stress (Arc et al., 2011).

In addition to its crucial role in the response to cold, carbohydrate metabolism appears essential in the transition from dormancy to active bud growth (**Figure 1**), as suggested by Park et al. (2009) and Signorelli et al. (2018). Changes in carbohydrate dynamics were linked to changes in dormancy status in sweet cherry with a degradation of starch into soluble sugars during dormancy onset and an increase in starch just before budburst (Kaufmann and Blanke, 2017). In parallel with starch dynamics, soluble sugars were shown to increase between autumn and winter followed by a significant decrease between winter and spring (Charrier et al., 2017). The bud capacity to burst is tightly linked to its supply in carbohydrates and as described previously, the carbohydrate uptake capacity increases in the bud after dormancy release with an increase in the expression and activity of plasma membrane transporters. Just before budburst, all sugars are transported in the sap toward the buds but during endodormancy, carbohydrate dynamics are restricted to the bud tissues. Tarancón et al. (2017) proposed that growth cessation and bud dormancy are consequences of carbon supply starvation syndrome linked to the sugar deficit. Over dormancy progression, and in response to winter conditions, soluble compounds (sucrose, glucose) are synthesized from the reserves accumulated during the growing season, such as starch grains (Felker et al., 1983; Guzicka, 2001; Xu et al., 2016; Guzicka et al., 2017). Interestingly, poplars overexpressing sucrose phosphate synthase – which accumulate more sucrose and starch than the wild-type poplars – are characterized by accelerated bud break, raising the possibility that enhanced sugar and/or starch reserves can promote accelerated dormancy breaking (Park et al., 2009). However, in the context of low carbon supply, it is possible that the gluconeogenesis pathway is activated to produce glucose from non-carbohydrate sources (**Figure 1**) as reported by Ruttink et al. (2007), associated with the generation of ROS. Similar pathway was described in dormant seeds (Einali and Valizadeh, 2017). In addition, a recent study showed that sucrose was synthesized during GA_4_-induced dormancy release (Zhuang et al., 2015), thus confirming the link between soluble sugars content and end of endodormancy.

During dormancy, glucose is metabolized in at least three pathways involved in the cell processes: the PPP leading to a detoxification system, glycolysis that synthesizes the pyruvate necessary for mitochondrial respiration, and fermentation producing lactate (**Figure 1**). We propose that the balance between all three pathways is key in the control of dormancy release.

## Hormonal Signaling

Phytohormones are plant molecules that are produced within the plant and control most, if not all, developmental aspects of plant life (Davies, 2013). ROS and hormones have been shown to act in an interdependent manner (Barba-Espin et al., 2010; Bahin et al., 2011; Oracz and Karpiński, 2016). For example, a recent study has shown this close link between the pathways with an inhibition of ROS formation by ABA, and a promotion of ROS formation by gibberellins (GAs) in seeds during cold stratification (Amooaghaie and Ahmadi, 2017), and conversely ROS mediate ABA and GA regulation through their catabolism and biosynthesis, respectively (Liu et al., 2010).

Hormonal pathways, including ABA, GAs, ethylene, auxin and cytokinins, have been demonstrated to be of great importance in the bud dormancy process (Mielke and Dennis, 1978; Rodríguez and Sánchez-Tamés, 1986; Wang et al., 1987; Piola et al., 1998; Ophir et al., 2009; Doǧramacı et al., 2013; Zhuang et al., 2013; Wen et al., 2016; Signorelli et al., 2018). Auxins were reported to be present at different concentration in buds throughout dormancy progression. Dormancy onset and cold treatment induces a reduction in auxin while quantity of auxins rises in ecodormancy until budburst as shown in hazelnuts (Rodríguez and Sánchez-Tamés, 1986) and grapevine (Aloni et al., 1990). It has been shown that auxins can be oxidized by two mechanisms in plants, a H_2_O_2-_dependent pathway, and a molecular dioxygen pathway, *via* peroxidases and membrane-bound NADPH oxidases (Pfeiffer and Höftberger, 2001; Kawano, 2003). Oxidation of auxin may thus yield ROS and be part of their generation during endodormancy progression. In addition, it has been shown in apple that genes related to auxin transport are major regulators of dormancy (Porto et al., 2015), and thus we could make the hypothesis that auxins may be involved to the propagation of the ROS signal through different territories, as this ROS signal propagation has been shown in grape buds during bud bursting (Meitha et al., 2015). Transcriptomic analyses of dormant buds suggest that brassinosteroid, salicylic-acid-, and jasmonic-acid-associated genes are differentially regulated during dormancy (Howe et al., 2015). The ethylene pathway is interesting when focusing on dormancy and oxidative stress signaling. Indeed, ethylene induced bud break in grapevine buds (Ophir et al., 2009) and low-temperature stress and HC treatment, closely linked to oxidative cues as stated before, provoke ethylene biosynthesis, associated with chilling requirement and dormancy release in peach and sweet cherry (El-Shereif et al., 2006; Del Cueto et al., 2017). In addition, the ethylene biosynthesis pathway, starting with ethylene precursors methionine and ACC, increases during endodormancy, resulting in the production of ethylene but also of hydrogen cyanide, therefore leading to increased levels of ROS (Ionescu et al., 2017). In seed dormancy, ABA and GAs act antagonistically thus it is not surprising that both pathways and their interaction have been closely studied in the context of bud dormancy (Rodríguez-Gacio Mdel et al., 2009). ABA has been demonstrated to promote shoot growth cessation and bud dormancy establishment (Le Bris et al., 1999; Guak and Fuchigami, 2001) whereas GA promotes growth and dormancy release (Rinne et al., 2011). In fruit trees, increases in bud ABA content have been reported at the beginning of endodormancy in the fall (Götz et al., 2014; Wang et al., 2016; Tuan et al., 2017; Li et al., 2018) followed by a rapid drop in response to cold (Leida et al., 2012) or dormancy-breaking agents (Seif El-Yazal et al., 2014; Zheng et al., 2015), accompanied by changes in the expression of genes related to ABA biosynthesis and degradation. Several studies showed that the expression of 9-*cis*-epoxycarotenoid dioxygenases (*NCED*), involved in ABA synthesis, is activated during dormancy induction and maintenance (Fennell et al., 2015; Wang et al., 2016; Chao et al., 2017; Li et al., 2018). In addition, after chilling requirements are satisfied, ABA levels decrease under the control of ABA 8′-hydroxylase (encoded by *CYP707A*), which is up-regulated during dormancy release (Zhang et al., 2015; Wang et al., 2016; Tuan et al., 2017; Li et al., 2018). Alternatively, inhibition of active ABA might be related to the production of conjugated forms of ABA that increases in response to cold temperatures: glucose ester of ABA (ABA-GE) in *Vitis* (Koussa et al., 1994) and an ABA-isomer in cherry (Götz et al., 2014). Interestingly, recent studies have indicated that early cultivars of Japanese apricot contained less ABA during dormancy than late cultivars (Wen et al., 2016), thus suggesting a dose-dependent control of dormancy. Interestingly, a close relationship between ABA and ROS has been shown not only during stomatal closure but also for seed dormancy. Notably, exogenous ROS application diminished ABA concentration in barley seeds during the after-ripening period (Wang et al., 1998), and increased its catabolism by up-regulating *CYP707A* genes in *Arabidopsis* during seed imbibition (Liu et al., 2010). Moreover, treatment of sunflower seeds during this after-ripening period enhances ROS production (Oracz et al., 2007), similarly to many studies including HC treatment on bud in various species. Thus, even though no studies have demonstrated these connections of ROS and ABA in buds, they are well known in various seed tissues. Therefore, as proposed by Leida et al. (2012), similar mechanisms may occur in bud dormancy. More precisely, we may compare the seed after-ripening period with bud endodormancy stage, while the imbibition process in seed may be similar to the ecodormancy period in buds. Nevertheless, the effect of ABA on bud dormancy is still not entirely understood, and ABA may control growth inhibition rather than play a direct role on dormancy regulation as suggested by Ramina et al. (1995) when they did not identify any strict relationship between ABA quantity and dormancy release in peach buds. Sensitivity to ABA may fluctuate as well, as shown in dormant pea seedling, where ROS inhibits the ABA signaling pathway during the imbibition period (Meinhard and Grill, 2001; Barba-Espin et al., 2010).

Gibberellic acids are particularly important as they may act in the growth renewal process during dormancy release. In fruit trees, several studies pointed out their implication in the control of dormancy progression, although few studies quantified them directly, but focused on their metabolism and on the effect of exogenous application. GA application may even substitute for chilling (Brown et al., 1960; Saure, 1985; Reinoso et al., 2002; Rinne et al., 2011; Zhuang et al., 2013) and GAs synthesis is promoted by dormancy breaking reagents (Seif El-Yazal et al., 2014). Nevertheless, the highest levels of GA_1_ and GA_3_ were found in dormant buds during endodormancy release and diminished afterwards (Luna et al., 1990; Wen et al., 2016). Overall, this is supported by expression analyses, with the up-regulation of *GA3-oxidase* (*GA3ox*) and *GA20-oxidase* (*GA20ox*), responsible for bioactive GA synthesis, under chilling treatment (Rinne et al., 2011) or around dormancy release (Bai et al., 2013; Wen et al., 2016). *GA2-oxidase* (GA2ox) genes, which encode the enzymes responsible for the deactivation of bioactive GA_4_ and GA_1_, are upregulated during dormancy and dormancy release in Japanese apricot buds (Yamane et al., 2008) but during ecodormancy as well in Japanese pear (Bai et al., 2013). Thus it appears that GAs regulation is tightly balanced between production and degradation, and they might enhance growth rate when the conditions are favorable. Interestingly, endogenous ROS application enhances endogenous GA concentration in *Arabidopsis* seed (Bahin et al., 2011) through diminution of its catabolism, and conversely GA_4_ application in apricot flower buds led to the development of oxidative stress and subsequent dormancy release (Zhuang et al., 2013). Thus there is obviously a tight link between ROS and GA, both influencing each other.

Taken as a whole, we can postulate that, similarly to seed dormancy, the hormonal balance between ABA and GA, which promotes dormancy and growth, respectively, may mediate the decision toward bud break, and as stated previously for different hormonal studies. Furthermore, as shown for the interaction between ethylene and ABA, ethylene modulating ABA degradation and signaling (Zheng et al., 2015), all hormonal pathways are interconnected and act together to control dormancy progression this balance may be directly influenced by ROS content, notably through redox control of the activity and symplasmic and apoplastic transport of plant growth regulator or transcriptions factors (Considine and Considine, 2016).

## Plasma Membrane and Cell Wall Modifications

**FIGURE 2:**
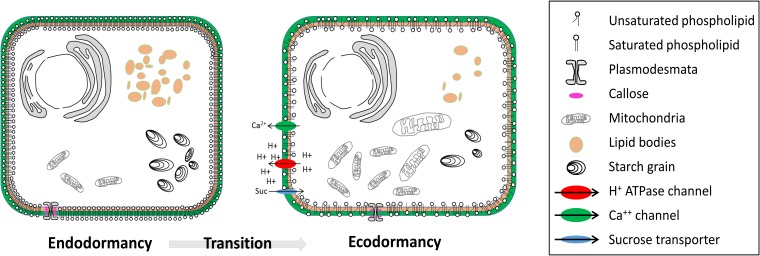
Cellular structure modifications from endodormancy to ecodormancy. During transition from endodormancy to ecodormancy in dormant buds, several structure modifications occur. The insaturation degree of plasma membrane lipids increases during transition from endo to ecodormancy. The number and size of starch grains and lipid bodies decrease during the transition from endo to ecodormancy. Conversely, the number and size of mitochondria increases during the transition from endo to ecodormancy. Transcripts of sucrose transporters, H+/ATPase channels and calcium channels increase during transition from endo to ecodormancy. The size of plasmodesmata increases during transition from endo to ecodormancy, as a result of net callose degradation.

On another scale, modifications of membrane structure may be influenced by ROS concentration, or may be responsible for changing metabolism and thus may enhance ROS production. For example, O_2_ diffusion through membranes may be more effective just before endodormacy release, inducing metabolism activity and then the increase of ROS production. On the other hand, as proposed above, ROS production may be caused by hypoxia. It is therefore essential to understand whether membrane modifications induce ROS production by raising metabolism as oxygen is more available, or if oxygen shortage generates ROS (Turrens, 2003). Molecular and metabolic changes associated with seasonal cycle of dormancy have been studied extensively in trees but structural changes at the cell level were less examined. Cells are organized in different compartments that enable their normal functioning. These compartments are delimited by membranes that are bilayers of complex lipids, partly permeable, associated with proteins (**Figure 2**). The stability of the membranes under cold stress highly depends on their functional and structural characteristics therefore the role of its components, lipids and proteins, is crucial. During winter, both dormancy and cold acclimation modify the cell structure and the two processes are usually difficult to separate. The lipid composition and membranous factors are modified during dormancy, notably to protect the cells from freezing-induced dehydration and lesions (Wang and Faust, 1990; Uemura and Steponkus, 1999). For example, it has been shown that the fluidity of the plasma membranes of bud cells in peach increases with chilling during dormancy release (Portrat et al., 1995), associated with a marked increase in total phospholipid content and in the relative level of linolenic acid (C18:3) (Erez et al., 1997). Moreover, low temperatures or thidiazuron, a growth regulator, increases the degree of unsaturation of fatty acids in the membrane lipids of apple buds, changes the polar head group composition, and changes sterol levels and composition (Wang and Faust, 1988, 1990). In addition, triggering dormancy release by GA_4_ application further confirmed that the composition in linoleic and linolenic acids are modified during dormancy progression (Zhuang et al., 2015).

Thus, winter cold temperatures lead to modifications in the membrane state as part of the cold acclimation process but also potentially the dormancy state. Nevertheless, no studies have been undertaken concerning the global lipid composition during the dormancy period with a lack of chilling, thus allowing a better understanding of the role of lipids over winter and spring rest. Studies investigating ultrastructure changes during dormancy are still rare but exceptions includes: plasmodesmata and lipid droplets analyses (Rinne and van der Schoot, 2004; Rinne et al., 2011; Paul et al., 2014) and cell wall thickening and an increase in vacuole precipitates during dormancy induction (Jian et al., 1997). Currently, no study has shown a direct relationship between ROS signaling and membrane modification in bud dormancy but analyses on seed germination have shown that hydroxyl radical OH- had a direct role on degradation involved in cell wall loosening (Müller et al., 2009). Therefore, we can hypothesize that ROS signaling may be involved in downstream cell wall loosening during bud dormancy release and growth resumption.

## Role of Membrane Bound Transporters

Apart from the changes in the saturation level of the lipids and types of lipids, other properties of the membranes are modified over dormancy progression (**Figure 2**). In particular, movements through the plasma membrane and long-distance transport change in response to chilling. In peach for example, active absorption of sucrose and other nutrients is stopped when the bud is in a state of deep dormancy while an active sucrose import was observed during growth resumption (Marquat et al., 1996). These modifications in cotransport H+/sucrose can be explained by structural changes of the plasma membrane (Wisniewski and Ashworth, 1986; Portrat et al., 1995), and more specifically by changes in ATPases activity (Giaquinta, 1979) (**Figure 2**). Plasma membrane H^+^ extrusion pumps (PM H^+^/ATPases) are key players in transport activity through their role in energizing solute transport (Alves et al., 2001) and studies revealed that H+/ATPases accumulation and activity are inhibited during endodormancy (Aue et al., 2000) but increase during endodormancy release or ecodormancy in peach (Gévaudant et al., 2001), walnut (Alves et al., 2007), and pear (Liu et al., 2012) (**Figure 2**). In the same time, the hydric status of buds varies characteristically with a marked dehydration in endodormancy and a water content increase in ecodormancy, just before budburst (Rinne et al., 1994; Yooyongwech et al., 2009; Götz et al., 2014; Kaufmann and Blanke, 2017). Water content was reported to increase in peach buds after a treatment with HC, a dormancy-breaking agent, suggesting that not only cold regulates the hydric status (Yooyongwech et al., 2012). The water status in buds is controlled by a range of membrane bound channels like aquaporins. Transcripts for two aquaporins showed differential spatiotemporal patterns in dormant peach buds in an interesting study by Yooyongwech et al. (2008). Their observations revealed that the activation of inter- and intra-cell communication through aquaporins resulted in a gradual increase in water content before growth resumption, which occurs earlier in low-chill cultivars than in high-chill cultivars.

Furthermore, connections between cells and organs over dormancy progression rely on the plasmodesmata functioning for carbohydrate and nutrient supply as well as signaling molecules. Plasmodesmata are not only essential for cell-to-cell transport, thus crucial for the bud functioning during dormancy, but they also control the supply route through the phloem between the buds and the shoot. Consequently, modifications of the transport activity by obstruction of the plasmodesmatal system lead to growth cessation, dormancy onset and dormancy release (Rinne and van der Schoot, 1998; Rinne et al., 2001; Xu et al., 2016). In their exhaustive review on plasmodesmata, Rinne and van der Schoot (2003) demonstrated the key role of plasmodesmata activity for symplasmic uncoupling and recoupling of vegetative bud cells during dormancy onset and dormancy release, respectively. In dormancy-inducing conditions, several studies have shown that callose (1,3-β-D-glucan) is deposited at the bottleneck region of plasmodesmata leading to diminished, if not stopped, transport and signaling between cells (Rinne et al., 2001). Net callose deposition is governed by the joint action of 1,3-β-glucansynthases and 1,3-β-glucanases (glucan hydrolase family 17, GH17), and shifts in the balance of these enzymes are central to the dormancy status and the growth potential of buds. Rinne et al. (2001, 2011) have hypothesized that the balance shifted toward net deposition as long as endodormancy lasts. Subsequently, during chilling-induced dormancy release, blocked plasmodesmata connections are restored within the bud by callose degradation. GAs seem to be implicated in the up-regulation of specific *1,3-*β*-glucanases* involved in orchestrating the chilling-induced callose breakdown to restore symplastic connections after endodormancy release (Rinne et al., 2011). By focusing on the ultra-structure of plasmodesmata Rinne et al. (2001) have shown that lipid bodies are targeted to the plasma membrane in close proximity to the plasmodesmata and may facilitate the restoration of plasmodesmata functionality (Rinne et al., 2011). Lipid droplets are membrane-bound storage organelles of universal occurrence. Recent analyses have suggested their role as signaling platforms that deliver proteins and signaling molecules (Murphy, 2012). In plants, they have been described as globules containing neutral lipids, triglycerids (TAG) or sterol esters, delimitated by a phospholipid monolayer (Farese and Walther, 2009; Chapman et al., 2012). van der Schoot et al. (2011) and Paul et al. (2014) have hypothesized that in buds, lipid bodies function as a vehicle that delivers proteins to the plasmodesmata, including 1,3-β-glucanases to the callose deposits in order to restore plasmodesmata function (Rinne et al., 2011). Overall, explaining the establishment and release of dormancy by the dynamics of callose and plasmodesmata is tempting but genetic and molecular evidence to support this hypothesis are still lacking and causality remains unproven. Recently, observations on spruce embryonic shoots have revealed that callose was still detected in plasmodesmata during ecodormancy (Guzicka et al., 2017). Furthermore, recent studies on architecture and permeability of plasmodesmata have shed light on the fine mechanisms regulating cell-to-cell connectivity. They show that, although callose is a central regulator of plasmodesmata, it does not necessarily mediate all changes to connectivity and to the size exclusion limit (SEL) for the molecules (Tilsner et al., 2016; Nicolas et al., 2017).

To our knowledge, no published study has uncovered interactions between ROS signaling and intercellular transport during bud dormancy. However, these links have been firmly suggested in other systems. For example, *Nicotiana benthamiana* mutants exhibiting increased production of ROS also displayed higher plasmodesmal transport (Stonebloom et al., 2012). In *Arabidopsis*, a study measuring root cell permeability also supports the hypothesis that ROS amounts regulate plasmodesmata function: application of low concentrations of H_2_O_2_ increased plasmodesmata permeability whereas high H_2_O_2_ concentrations induced plasmodesmata closure (Rutschow et al., 2011). These results suggest that ROS may participate in promoting the formation and alteration of plasmodesmata, or callose deposit, thus controlling communication and transport. Rutschow et al. (2011) speculate that this signaling is linked to differential stress response: low amounts of ROS indicate mild stresses that might be mitigated by increased cellular transport while the response to extreme stressed revealed by acute ROS signals necessitates cellular isolation. For bud tissues, the question here is therefore which amount of ROS is produced during dormancy progression and release and how this signal is transduced for cellular transport.

## Conclusion

**FIGURE 3:**
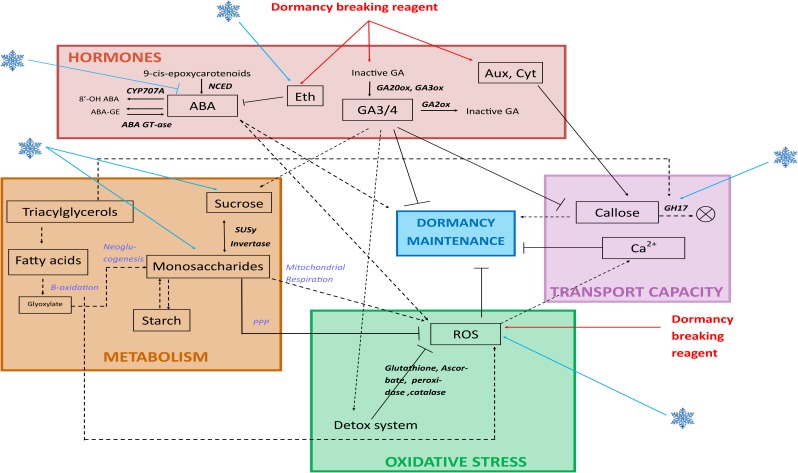
Dormancy pathways and their interactions. During dormancy, several pathways have been shown to have a role and relate to each other. **Hormones**: abscisic acid (ABA) turnover is regulated through action of 9-*cis*-epoxycarotenoid dioxygenases (NCED) genes which are repressed by the action of cold temperatures, and is involved in dormancy maintenance and reactive oxygen species (ROS) generation. Ethylene production is under control of cold temperatures (blue flake) and dormancy breaking reagents, and is involved in ABA diminution. Active gibberellic acids (GA) are produced by GA20ox and GA3ox, and are involved in dormancy alleviation and favourise the detoxification system; they are inactivated by GA2ox. Auxins and cytokinins act through enhancing callose deposition at plasmodesmata. **Transport capacity**: callose deposition at plasmodesmata is involved in dormancy maintenance; Glycoside Hydrolases 17 (GH17) are involved in digesting callose. Cold temperatures enhance GH17 expression. Calcium channels inhibit dormancy maintenance. **Metabolism**: cold temperatures enhance sucrose and monosaccharides concentration. Monosaccharides are also produced from the beta-oxidation and neoglucogenesis from fatty acids, and these monosaccharides produce ROS *via* mitochondrial respiration or are oxidized *via* the Pentose Phosphate Pathway (PPP) and participate to ROS detoxification. beta-oxidation produces ROS. **Oxidative stress**: cold temperatures (blue flake) and dormancy breaking reagents enhance ROS production, and ROS production inhibits dormancy maintenance.

Oxidative stress, carbohydrates metabolism highly linked to the mitochondrial respiration, hormones and transport capacity associated to plasma membrane and cell wall properties have been shown to play important roles in bud dormancy process. As these pathways interact between them, dormancy mechanism is very complex. The main pathways involved in bud dormancy are synthesized in **Figure 3**. Overall, these studies highlight the pivotal role of ROS production and detoxification systems for dormancy release. As a consequence of a combination of stresses and the decrease of cell metabolism, dormant buds accumulate ROS and their removal by scavenging and detoxification systems seems to be associated with breaking of dormancy. Implication of these mechanisms is further validated by the action of HC, potentially linked to the generation of sub-lethal oxidation stress. The question remains whether slow accumulation of ROS, as a consequence of winter temperatures and low metabolism, triggers dormancy release when a sub-lethal threshold is reached, or a prompt shift into stress-inducible conditions leads to a dormancy-alleviating response. In this case, the whole concept of chilling requirements could be questioned. Further studies are necessary to address these hypotheses, including the question of toxicity of dormancy-breaking treatments, in relation to ROS production.

## Author Contributions

RB, BW, and ED designed the manuscript. RB and BW wrote the manuscript. BW and ED critically evaluated the manuscript.

## Conflict of Interest Statement

The authors declare that the research was conducted in the absence of any commercial or financial relationships that could be construed as a potential conflict of interest.
